# Arabinogalactan proteins answer a great puzzle

**DOI:** 10.1016/j.tcsw.2025.100142

**Published:** 2025-04-25

**Authors:** Derek T.A. Lamport

**Affiliations:** School of Life Sciences, University of Sussex, Falmer, Brighton BN1 9QG, UK

Sixty five years after their discovery) hydroxyproline-rich proteins of the cell surface (Lamport and Northcote, 1960), extensins and arabinogalactan proteins (AGPs) remain somewhat mysterious (Silva et al., 2020). However, relatively unappreciated classical AGPs open a new chapter in plant biology.

Mature sciences like plant biology rarely yield dramatic breakthroughs. Indeed, the last plant scientist won a Nobel Prize more than forty years ago for transposable elements (Mcclintock, 1983) and forty five years ago the Nobel Prize for proton pumps (Mitchell, 1961, Mitchell, 1978).

Slow progress since then has left many unresolved questions in areas of cell wall, polysaccharide structure and function summarised recently by several leading plant cell wall researchers who pose *“five unanswered questions”* (Boerjan et al., 2024) inviting the corresponding response: **What questions have been answered since Mitchell and McClintock?** Here, classical arabinogalactan glycoproteins (AGPs) answer several questions about growth and development, particularly plant movements (tropisms including gravitropism Lamport, 2022) that involve oscillatory growth typically of pollen tubes (Roy et al., 1999), circumnutation of tendrils (Darwin, 1880) shoots and root tips (Taylor et al., 2021). More recent authors suggest that it depends on an internal oscillator of unspecified mechanism (Brown, 1991).The precise mechanism is “A Great Puzzle” but a missing piece of the puzzle emerged recently with the discovery of specific AGP—Ca^2+^ binding at the cell surface (Lamport and Varnai, 2013) but overlooked with occasional exceptions comprehensively reviewed by (Silva et al., 2020)**.** The inferred **underlying growth oscillator** (Lamport and Varnai, 2013; Lamport, 2023) **involves four components embedded in the plasma membrane: AGPs, proton pumps, Ca**^**2+**^ **channels and auxin efflux “PIN” proteins:** Meristematic cell auxin efflux activates proton pumps that dissociate AGP—Ca^2+^ to supply Ca^2+^ channels and generate cytosolic Ca^2+^ oscillations ubiquitous in plant cells (Calder et al., 1997). Thus AGP—Ca^2+^ answers key questions about the plasma membrane proton pump (Li et al., 2022) and its hitherto unknown major role Lamport and Varnai, 2013) in generating cytosolic Ca^2+^ oscillations and thus Ca^2+^ homeostasis which regulates cytosolic enzymes that activate exocytosis (Roy et al., 1999) required for growth:

While auxin activates the proton pump the periodicity of its H^+^-ATPase activity is a complex function of several variables chiefly, the auxin supply via “PIN” proteins and proton pump H^+^-ATPase activation by a complex auxin signalling cascade; arguably that involves association of the auxin binding protein (ABP1) with the transmembrane kinase (TMK-1) that phosphorylates the proton pump and is thus its ultimate activator (Friml et al., 2022).

It is also a crucial weak spot in plant defence exploited both by pathogens and human agency. Thus, continuous maximal activation of the pump by the fungal toxin fusicoccin (De Michelis et al., 1988) and the auxin analogue 2,4-D weed killer cause unregulated growth and self-destruction.

The diverse role of protons is a universal theme including both abiotic stellar evolution and biotic planetary evolution (Miller, 1953) where mitochondrial proton pumps generate ATP that energise life (Mitchell, 1961). However, quite remarkably, ATP reversal of the plasma membrane proton pump generates extracellular proton gradients (Bonner, 1934) with so-called “acid growth” of plants possibly a direct effect of secreted protons on cell wall rheology (Cosgrove, 2023). However, an alternative scenario suggested here, involves AGP—Ca^2+^ dissociation (Lamport, 2024) previously overlooked as the major source of cytosolic Ca^2^ rather than the ER. Thus proton-dependent Ca^2+^-homeostasis perhaps began in Darwin's warm little pond (Follmann and Brownson, 2009) with photosynthetic proton gradients energised by visible light initially photosystem-I. Later acquisition of the photosystem-II proton pump photolysis of water (Hill and Bendall, 1960) and concomitant release of oxygen had enormous ramifications. That includes the direct fixation of molecular oxygen into proline as the source of hydroxyproline shown in a classic paper (Lamport, 1963). Thus, proline hydroxylation catalysed by prolylhydroxylase with oxygen as a co-substrate is the source of hydroxyproline and the extracellular matrix of plants and animals thus enabling multicellular evolution. In animals intermolecular hydroxyl H-bonds of hydroxyproline (Hyp) stabilise the triple helix of collagen an essential component of cartilage and bone and thus, plays a pivotal role in morphogenesis. In plants the hydroxyl group involves a crucial glycopeptide linkage that anchors glycosubstituents. Hyp-O-arabinosylation stabilises extensin networks, while Hyp-O-linked arabinogalactan glycomodules bind AGP-Ca^2+^as the cell surface reservoir for Ca^2+^homeostasis. These roles of the hydroxyproline hydroxyl testify to Nature's parsimony and answer significant questions about the role of AGPs widely relevant to climate change (Lamport, 2024). Thus AGPs, like the proverbial elephant in the room, guard a well-hidden puzzle of Nature: An AGP key unlocks components of the growth oscillator and Ca^2+^ homeostasis regulation of plant growth.

## CRediT authorship contribution statement

**Derek T.A. Lamport:** Writing – review & editing, Writing – original draft, Conceptualization.

## Declaration of competing interest

The authors declare that they have no known competing financial interests or personal relationships that could have appeared to influence the work reported in this paper.

## Data Availability

No data was used for the research described in the article.
